# Colorectal cancer in Iran: Epidemiology and morphology trends

**DOI:** 10.17179/excli2016-346

**Published:** 2016-11-28

**Authors:** Hosein Rafiemanesh, Reza Pakzad, Mehdi Abedi, Yones Kor, Jalal Moludi, Farhad Towhidi, Behnam Reza Makhsosi, Hamid Salehiniya

**Affiliations:** 1 Students' Research Committee, School of Public Health, Shahid Beheshti University of Medical Sciences, Tehran, Iran; 2 Students' Research Committee, Ilam University of Medical Sciences, Ilam, Iran; 3 School of Medicine, Islamic Azad University Tehran Medical Branch, Tehran, Iran; 4 Department of Elder Nursing, School of Nursing and Midwifery, Iran University of Medical Sciences, Tehran, Iran; 5 Department of Biochemistry and Diet Therapy, Faculty of Nutrition, Nutrition Research Center, Tabriz University of Medical Sciences, Tabriz, Iran; 6 Imam Reza Hospital, Kermanshah University of Medical Sciences, Kermanshah, Iran; 7 Zabol University of Medical Sciences, Zabol, Iran; 8 Tehran University of Medical Sciences, Tehran, Iran

**Keywords:** colorectal cancer, epidemiology, morphology, trend, Iran

## Abstract

Colorectal cancer is one of the most prevalent cancers in different countries, including Iran. No comprehensive study has been done in the country for colorectal cancer, but information on the incidence and trends is essential to planning. This study aimed to evaluate the occurrence and morphology of colorectal cancer and its trend in Iran. This study was conducted using data from the national cancer registry system in Iran from 2003-2008. We used joinpoint regression analysis for assessing incidence time trends and morphology change percentage. Of all cases of colorectal cancer, 61.83 % were colon cancer, 27.54 % rectal cancer, 7.46 % rectosigmoid cancer, and 3.10 anal cancer. The most common histological types with the frequencies of 80.85 % was related to adenocarcinoma, NOS. The Annual percentage changes (APC) in ASIR for colorectal cancer significantly increased in both men and women. APC in ASIR was 13.7 (CI: 10.5-17.1) in women and 16.4 (CI: 12.4-20.5) in men. APC of adenocarcinoma in villous adenoma showed significant declining trend (p<0.05), while APC of adenocarcinoma, NOS had a constant trend. The incidence of the cancer in recent years has increased in Iran because of changes in lifestyle and diet. Therefore, further studies are necessary to detect the cause of this cancer and perform preventive measures.

## Introduction

Colorectal cancer is one of the most common cancers in the world. It includes 9 % of all cancers (Haggar and Boushey, 2009[13]). This cancer is the second common cancer and the fourth leading cause of cancer death in the world (Glade, 1999[10]; Jemal et al., 2011[15]; Rafiemanesh et al., 2016[29]). During recent decades, the incidence of colorectal cancer has been a great increase in the world. In 1990, the number of new cases was 783,000 (401,000 men and 381,000 women (Boyle and Langman, 2000[3])). However, in 2012, 1,361,000 new cases of colorectal cancer (746,000 men and 614,000 women) occurred (Ferlay et al., 2015[7]). The incidence of colorectal cancer is different in the world about 20 times, and most of cases are available in Western industrial countries. The highest incidence of colorectal cancer was seen in Australia and New Zealand (39 per hundred thousand), West Europe (33.1 per hundred thousand), North America (30.1 per hundred thousand), and in East Asia, especially in Japan (18 per hundred thousand). Colorectal cancer was the lowest in Africa (3.6 per hundred thousand) and South Asia, including India and China, (3.6 per hundred thousand) (Boyle and Langman, 2000[3]; Potter and Hunter, 2009[26]). The mortality rate for colorectal cancer is also high worldwide, and 394,000 cases annually die from the cancer (Boyle and Langman, 2000[3]; Haggar and Boushey, 2009[13]). Most of colorectal cancer mortality was in central European countries (in men and women 20.3 and 12.1 per hundred thousand, respectively), and the lowest in Middle Africa (in men and women, 3.5 and 2.7 per hundred thousand, respectively) (Miladinov-Mikov, 2010[22]). Although colorectal cancer is common in both sexes, the incidence is higher in men than women, and the standardized incidence of colorectal cancer was in men 20.6 % and in women 14.6 % per hundred thousand (Ferlay et al., 2015[7]). The incidence of colorectal cancer increases with age, so that the highest incidence is observed after 75 years and above (Boyle and Leon, 2002[4]). 

Colorectal cancer is one of the most common cancers in Iran. This cancer is the third most common cancer in Iranian men (standardized incidence: 8.1-8.3 per 100,000) and the fourth most common cancer in women with a standardized incidence of 6.5 to 7.5 per 100,000 (Moghimi-Dehkordi et al., 2008[23]; Kolahdoozan et al., 2010[18]). It is a multi-factorial disease. Low physical activity, high BMI, high-fat diet, alcohol consumption, low intake of vegetables and fruit, tobacco smoking, a family history, and use of certain medications, including Contraception pills and non-steroidal anti-inflammatory drugs (NSAIDs), are risk factors for the cancer (Graham et al., 1978[12]; Giovannucci et al., 1995[9]; Boyle and Leon, 2002[4]; Hosseini et al., 2004[14]; Johnson et al., 2013[16]).

Some studies have showed changes in the epidemiology and morphology trends of the cancers in some countries and Iran (Koohi et al., 2014[19]; Zahedi et al., 2014[36]; Almasi et al., 2015[1]; Keyghobadi et al., 2015[17]; Pakzad et al., 2015[25]; Razi et al., 2015[31]; Rafiemanesh et al., 2014[28], 2016[27]; Salehiniya et al., 2016[32]).The incidence and mortality of colorectal cancer is declining in advanced Western countries (Siegel et al., 2014[33]), while the incidence of the cancer in recent years has increased in Iran because of lifestyle changes, dietary changes, reducing physical activity and improving diagnostic techniques. Malekzadeh et al. (2009[20]) also showed that the incidence of the cancer has increased in both sexes during the last decade. No comprehensive study has been done in the country for colorectal cancer, but information on the incidence and trends is essential to planning. Therefore, this study aimed to evaluate the occurrence and morphology of colorectal cancer and its trend in Iran. 

## Materials and Methods

### Data source

This study was conducted using (published) data from the national cancer registry system and Disease Control and Prevention (CDC) report of ministry of Health and Medical Education in Iran from 2003-2008 (Goya, 2007[11]). More detail about cancer registry previously published (Razi et al., 2015[30]).

Data were collected retrospectively reviewing all new colorectal cancer patients in Cancer Registry Center report of health deputy for Iran during a 6-year period (2003-2008), based on The International Classification of Diseases for Oncology (ICD-OC: topography with ICD-OM: morphology) Colorectal cancer was defined as ICD-O C18-21(WHO, 2013[35]). ICD-O in comparison with ICD-10, also describes the type or morphology of the neoplasm. This study investigated all cases of the morphology of Adenocarcinoma, NOS (M-8140/3), Mucinous & mucine producing adenocarcinoma (M-8480/3 & 8481/3) and adenocarcinoma in villous Adenoma (M-8261/3).

### Statistical analysis

We calculated crude incidence rate (CIR) and the age-standardized incidence rate (ASIR) per 100,000 persons. We used direct standardized method using world standard population (Dos Santos Silva, 1999[6]). To describe incidence and age group incidence time trends, we use age-standardized incidence rate (ASIR) and carried out Joinpoint regression analysis using the software Joinpoint Regression Program, Version 4.2.0.2 June 23, 2015. 

So to analyse morphology change percentage trends of colorectal cancer for six year, we carried out Joinpoint regression analysis using the software Joinpoint Regression Program. 

Joinpoint regression analysis involves fitting a series of joined straight lines on a log scale to the trends. The aim of the approach is to identify possible joinpoints where a significant change in the trend occurs. The final model selected was the most parsimonious of these, with the estimated annual percent change (APC) based on the trend within each segment. In describing trends, the terms “significant increase” or “significant decrease” signify that the slope of the trend was statistically significant (P < 0.05). All reported P-values are two-sided.

## Results

During the six-year study, 25,704 cases of colorectal cancer were registered. Of all cases 44.27 % (11,380 cases) were women and 55.73 % (14,324 cases) men. The male to female sex ratio was 1.26. Of all cases of colorectal cancer, 61.83 % (15, 892 cases) were colon cancer (C18), 27.54 % (7,080 cases) rectal cancer (C20), 7.46 % (1,918 cases) rectosigmoid cancer (C19), and 3.10 (796 cases) anal cancer (C21). The most common histological types with the frequencies of 80.85 % and 8.96 % were related to adenocarcinoma, NOS (M-8140/3) and mucinous and mucine producing adenocarcinoma (M-8480/3 & 8481/3). 

### Epidemiological trend

Age standardized incidence rate (per 100,000) increased for colorectal cancer in women from 5.47 to 11.12 and in men from 5.56 to 12.7 between 2003 and 2008 (Table 1(Tab. 1)). 

**Figure 1:**
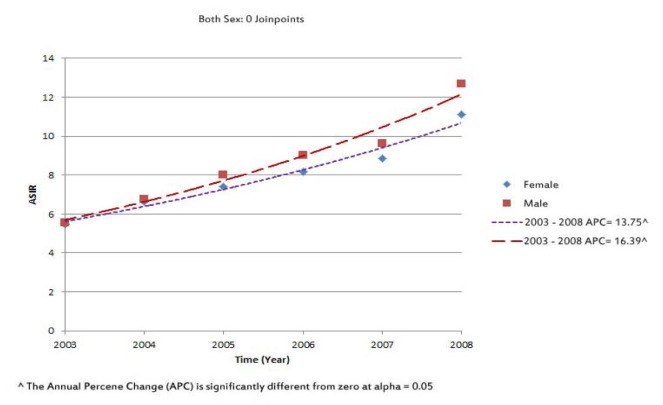
Trends of the standardized incidence rate of colorectal cancer by sex in Iran, 2003-2008

The Annual percent changes (APC) in ASIR for colorectal cancer significantly increased in both men and women. APC in ASIR was 13.7 (CI: 10.5-17.1) in women and 16.4 (CI: 12.4-20.5) in men (Figure 1(Fig. 1)).

### Histological trend

Three histological types of adenocarcinoma, NOS and mucinous and mucine producing adenocarcinoma included 90.12 % in women and 89.56 % in men from all total histology of colorectal cancer. The histology type of adenocarcinoma in villous adenoma (M-8261/3) included 0.5 % women and 0.56 % men from all types of the cancer. During the study, the percentage allocated to each of histology was significantly different (Table 2(Tab. 2)).

APC of adenocarcinoma in villous adenoma showed significant declining trend (p<0.05), while APC of adenocarcinoma, NOS had a constant trend. Other types (mucinous and mucine producing adenocarcinoma) showed a decreasing trend, but these changes were not significant (p>0.05) (Table 3(Tab. 3)).

## Discussion

According to our findings, the incidence of colorectal cancer had increased in both sexes between 2003 and 2008 (in women from 3.92 to 7.78 and in men from 5.56 to 12.7 per 100,000). The increased incidence of colorectal cancer was also seen in the world (in 1985, 16.6 in men and in women 14 per 100,000, and in 2012 in men 20.6 and in women 14.7 per 100,000) (Boyle and Langman, 2000[3]; Hosseini et al., 2004[14]). However, the incidence of colorectal cancer is lower in Iran than other countries, but it is expected to be a growing trend in the future (Malekzadeh et al., 2009[20]). In the same period, Australia with an incidence of 39 per hundred thousand, countries of West Europe with 33.1 per hundred thousand, North America with an incidence of 30.1 per hundred thousand and Japan with 18 per hundred thousand had the highest incidence of colorectal cancer in the world (Boyle and Langman, 2000[3]; Potter and Hunter, 2009[26]). 

One reason for the increased incidence of colorectal cancer in Iran can be changes in lifestyle and diet. Western diet with high fat and low fiber is directly related to colorectal cancer (Graham et al., 1978[12]). Studies have shown that per capita consumption of fat has increased in Iran (Azizi et al., 2001[2]; Ghassemi et al., 2002[8]). Also, the prevalence of BMI in different age groups over the past decade has been an upward trend in Iran (Djazayery and Pajooyan, 2000[5]; Azizi et al., 2001[2]). The prevalence of fast foods that is a Western diet over the past years has been increasingly frequent in Iran (Hosseini et al., 2004[14]). All above factors may be reasons of the increased incidence of colorectal cancer in Iran.

Smoking and reduction of physical activity are the reasons that may explain the increased incidence of colorectal cancer. Evidences showed that smoking has increased in the past few decades in Iran (Meysamie et al., 2010[21]). Hosseini (2004[14]) revealed that Iran's traditional lifestyle is changing to the modern Western lifestyle (along with reduction in physical activity and an increase in consumption of cigarettes).

Another reason that may increase the risk of colorectal cancer is an increase in life expectancy. Although the study did not examine the incidence of cancers in the highest age group, a study introduced 75 years and above as the peak of incidence of colorectal cancer (Moghimi-Dehkordi et al., 2008[23]); while Hosseini (2004[14]) expressed the age group of 50 to 60 and Siegel (2014[33]) the age group of 65 to 79 years as the age groups with the highest incidence of colorectal cancer. These findings show the effects of age on the incidence of colorectal cancer. The availability of diagnostic services and treatment can cause differences in the incidence of colorectal cancer in developed countries compared with developing countries. With increasing development, diagnostic services including colorectaloscopy and ultrasound, will be more accessible, and therefore the chance of cancer diagnosis increases (Ward et al., 2004[34]). 

In our study, over 55 % of cases were males. Other studies indicated that the incidence is higher in men (Miladinov-Mikov, 2010[22]; Haggar and Boushey, 2009[13]; Potter and Hunter, 2009[26]; Siegel et al., 2014[33]). The sex ratio of men to women in our study was 1.26. Miladinov-Mikov (2010[22]) reported the sex ratio of 1.4, similar to our findings.

In terms of morphology, we can say that in both sexes, the most common type was adenocarcinoma. Mucinous and mucine, producing adenocarcinoma, and villous Adenoma were other ranks. Nawa (2008[24]) showed that adenocarcinoma was more than other types, and included 83 % of all cases. 

In our study, most cases suffered from colorectal cancer, so that 61 % of the anatomical location of the cancer was the colorectal. Siegel also stated that more than 66 % of the anatomical location of the cancer was the colorectal (Siegel et al., 2014[33]). In another study, it was also found that approximately 50 % of colorectal cancer cases had involvement in horizontal and ascending colorectal (Hosseini et al., 2004[14]). However, in colorectal cancer cases with the proximal colorectal is increasing due to the widespread use of diagnostic techniques such as colorectaloscopy (Nawa et al., 2008[24]; Haggar and Boushey, 2009[13]). 

## Conclusion

Our findings revealed that the trend of colorectal cancer in Iran is rising which could be due to lifestyle changes, an increase in smoking, reduction in physical activity, and poor diet. According to the heavy burden of colorectal cancer on the health system and a waste of cost, measures should be taken to reduce the disease, prevention, and early diagnosis of the disease. More studies are also useful to find the cause of the issue.

## Conflict of interest

The authors have no conflict of interest.

## Figures and Tables

**Table 1:**
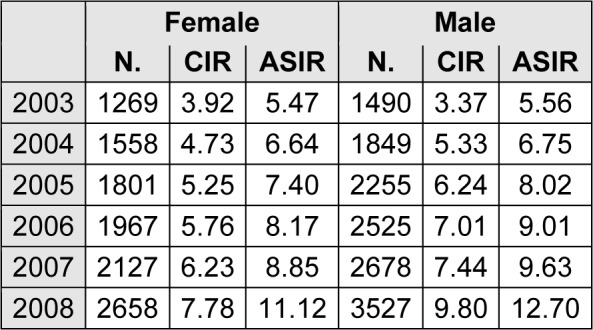
Number of cases, crude incidence rate and age-standardized incidence rates (per 100,000) of colorectal cancer by sex in Iran, 2003-2008

**Table 2:**
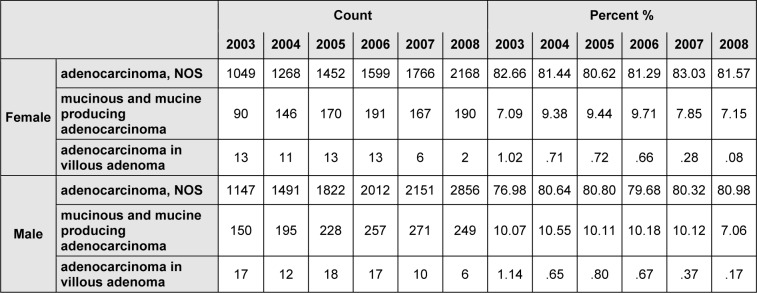
Number and percentage allocated of three common colorectal cancers morphology in Iran, 2003-2008

**Table 3:**
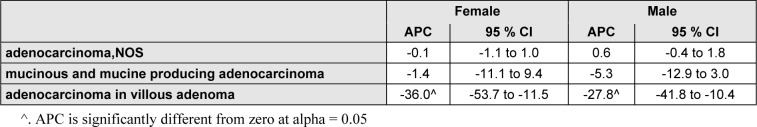
Joinpoint analyses of cancers percentage allocated to the morphology data for colorectal cancer in Iran, 2003-2008
